# Adaptive Dimensionality Reduction with Semi-Supervision (AdDReSS): Classifying Multi-Attribute Biomedical Data

**DOI:** 10.1371/journal.pone.0159088

**Published:** 2016-07-15

**Authors:** George Lee, David Edmundo Romo Bucheli, Anant Madabhushi

**Affiliations:** 1 Case Western Reserve University, Department of Biomedical Engineering, Cleveland, OH, United States of America; 2 CIM@LAB Research Group, Universidad Nacional de Colombia, Bogota, Colombia; Nanjing University of Aeronautic and Astronautics, CHINA

## Abstract

Medical diagnostics is often a multi-attribute problem, necessitating sophisticated tools for analyzing high-dimensional biomedical data. Mining this data often results in two crucial bottlenecks: 1) high dimensionality of features used to represent rich biological data and 2) small amounts of labelled training data due to the expense of consulting highly specific medical expertise necessary to assess each study. Currently, no approach that we are aware of has attempted to use active learning in the context of dimensionality reduction approaches for improving the construction of low dimensional representations. We present our novel methodology, AdDReSS (Adaptive Dimensionality Reduction with Semi-Supervision), to demonstrate that fewer labeled instances identified via AL in embedding space are needed for creating a more discriminative embedding representation compared to randomly selected instances. We tested our methodology on a wide variety of domains ranging from prostate gene expression, ovarian proteomic spectra, brain magnetic resonance imaging, and breast histopathology. Across these various high dimensional biomedical datasets with 100+ observations each and all parameters considered, the median classification accuracy across all experiments showed AdDReSS (88.7%) to outperform SSAGE, a SSDR method using random sampling (85.5%), and Graph Embedding (81.5%). Furthermore, we found that embeddings generated via AdDReSS achieved a mean 35.95% improvement in Raghavan efficiency, a measure of learning rate, over SSAGE. Our results demonstrate the value of AdDReSS to provide low dimensional representations of high dimensional biomedical data while achieving higher classification rates with fewer labelled examples as compared to without active learning.

## 1 Introduction

The ability to mine disease patterns from large biomedical datasets could enable the identification of prognostic disease markers, which in turn, could save lives, reduce morbidity, and alleviate the overall cost of healthcare today. Generally speaking, biomedical data may be regarded as a collection of diagnostic attributes, which can be obtained from a variety of sources, ranging from medical imagery, DNA microarrays, or protein expression data obtained from mass spectrometry techniques [1–6]. Most popular approaches to identify disease patterns are some variant of supervised classification strategies. In these approaches, classifiers are taught to distinguish between disease classes via a collection of these attributes and labeled training instances [1].

One of the primary challenges in building predictors for biomedical data is that it is typically high dimensional (large *K*) with relatively few samples (small *N*) [7]. Particularly in the case of DNA microarray data, the number of features can number in the tens of thousands [8]. Machine learning classifiers are often used to leverage the predictive power of a multitude of features and discriminate between patients with different underlying pathologies [2–6]. However, given the ‘curse of dimensionality’ problem [9], where *K* > *N*, it can be difficult to build a generalizable classifier from biomedical data. The Hughes effect [10] states that given a fixed number of training samples, increasing the dimensionality eventually reduces the predictive power of a classifier. This is because training a discriminating classifier in a high dimensional feature space results in many potential class separation boundaries for distinguishing the instances to be classified [11]. This implies that before these measurements can be incorporated within a classifier to generate predictions, the original measurements need to be first reduced to a smaller number of variables, *K* < <*N*, in order to build an accurate and generalizable classifier.

In the case of very high dimensional data, it has been desirable to represent the data in a low dimensional representation that can allow for the classes to be separable. [3, 12–14]. Feature selection is one method to reduce dimensionality by identifying the best *k* < <*K* features to represent the data [14–18]. While more readily interpretable compared to dimensionality reduction methods, feature selection methods may not provide the most compact or efficient low dimensional representation due to curse of dimensionality and possible presence of redundant and correlated features.

Dimensionality reduction (DR) methods, such as Principal Component Analysis (PCA) [19], have been used for analyzing high dimensional biomedical data [3] by mapping high dimensional data into a low dimensional embedded representation (or embedding). DR methods help to mitigate the ‘curse of dimensionality’ problem by learning a low dimensional representation which aims to approximate the original high dimensional features with fewer variables. DR methods can be grouped into two broad classes: linear and nonlinear methods.

Linear DR methods such as PCA, Linear Discriminant Analysis (LDA) [11], and Multidimensional Scaling (MDS) [20, 21], generally preserve Euclidean distances when mapping data into the embedding space. For example, PCA [19] determines the optimal projections of the data by a rotation of the high dimensional space to the axis of greatest variance. Alternatively, in MDS [20], Euclidean distances between each pair of data points are collected into a pairwise affinity matrix, which is then mapped into a low dimensional embedding which best preserves these distances.

In contrast, nonlinear dimensionality reduction (NLDR) methods [22–25] are founded on the premise that Euclidean distance does not represent true object similarity. In fact, researchers have found that NLDR methods may be better suited towards classification of high dimensional biomedical data compared to linear DR methods [12, 13, 26]. Graph-based DR methods such as Graph Embedding [23] are similar to the idea of manifold learning [24], where a graph dictates similarity between data points via a set of weighted edges. The graph itself is representative of an abstract low dimensional manifold which encompasses all data points and is embedded in the high dimensional space [24]. In order to extract the manifold from the high dimensional space, similarity between data points is re-defined as distances along the graph and the graph distance information can be projected into a low dimensional embedding. Specifically, NLDR methods such as Isomap [24], Locally Linear Embedding (LLE) [25], and Graph Embedding (GE) [23] have been shown to provide low dimensional data representations for improving classification performance and overall data interpretation [12, 13].

While unsupervised methods, such as NLDR schemes, have been utilized for preliminary analysis of data, for classification tasks, it is desirable to incorporate all available object class labels to optimize the embedding for class separation, as opposed to basing the affinities solely based off the pre-defined similarity criterion [27–29]. Recently, there has been a great deal of interest in semi-supervised dimensionality reduction (SSDR) methods, which utilize labeled instances to improve separation of object classes in the low dimensional embedding [30–36]. This is typically done by extending the pairwise affinity matrix of previous DR methods to incorporate class label information, such that if a pair of objects belong to same class, they are weighted to be more similar and will be mapped to be closer together in the low dimensional embedding. Similarly, if a pair of objects are of different classes, they are weighted to be less similar and will be mapped farther away in the embedding. Sugiyama et al. [33] applied semi-supervised learning (SSL) to Fisher’s discriminant analysis in order to find the linear projection that maximized object class separation. Verbeek et al. [37] utilized a method for semi-supervised learning using Gaussian fields with locally linear embedding for object pose recognition. Yang et al. [34] similarly applied SSL toward manifold learning methods. Zhao [35] presented a semi-supervised method for graph embedding which utilizes weights to simultaneously attract samples of the same class labels and repel samples of different class labels given a neighborhood constraint. Zhang [36] employed a similar approach to SSDR as Zhao, but without utilizing neighborhood constraints.

In addition to the large-*K*/small-*N* problem, a second challenge with building predictors for biomedical data is that very often, biomedical datasets are not adequately labeled or annotated [38]. This is due to the significant overhead involved in procuring well-annotated biomedical datasets and also due to the fact that invariably an expert is required to perform this task [5]. Hence, if one is attempting to build a predictor to identify disease aggressiveness or predict long term outcome in a patient, one would need a well curated and annotated dataset to provide training instances for the predictor. Active learning (AL) can reduce the number of samples needed to train an accurate predictor.

AL is a specific instance of semi-supervised learning, where the learning algorithm may interactively query the desired labels from a user or other source [39]. AL differs from random sampling, which queries training instances randomly from an unlabeled pool [40]. The objective of AL is to find an optimal training set. The benefits of using AL are twofold as 1) classifier accuracy can be improved, and 2) the number of training labels necessary to achieve a classification goal is reduced.

While AL has been used for providing fewer, optimal instances for training a classifier, its extension towards learning the best training instances for improving the quality of low dimensional embedding representations has not been heavily investigated [37, 41]. Zhang et al. [42] has suggested that searching in a locally linear or manifold space could provide more representative points for active learning. Thus, an extension of AL to SSDR would be important for prediction and representation of biomedical data.

In this paper, we present a novel dimensionality reduction (DR) method, AdDReSS (Adaptive Dimensionality Reduction with Semi-Supervision), which aims to seamlessly integrate semi-supervised dimensionality reduction and active learning. This allows AdDReSS to construct low dimensional data representations to improve classification of high dimensional biomedical data while using fewer labels compared to previous SSDR methods.

The major contributions and implications of this work are: First, a novel NLDR method which seamlessly incorporates active learning and semi-supervised learning to guide embedding construction. Second, a demonstration showing the effects of active learning towards improving embeddings generated via SSDR compared to random sampling. Third, a simple framework that could be extensible for other SSDR methods to create more discriminatory low dimensional representations.

We evaluated our methodology on different tasks for four relevant medical datasets: (a) Discrimination of tumoral and non-tumoral prostate samples in a gene expression dataset [8], (b) Discrimination of neoplastic and non-neoplastic disease within the ovary in a protein expression dataset [4], (c) Mitosis detection in breast cancer images [43], and (d) Identifying white matter and grey matter in a Brain MR Imaging dataset [44]. These datasets were chosen to represent varied types of imaging and non-imaging biomedical data—radiologic medical imaging, histologic imaging, DNA microarray, and proteomic spectra.

The rest of this paper is organized as follows. In Section 2, we formalize notation and provide an overview of an unsupervised dimensionality reduction method (Graph Embedding) and a semi-supervised dimensionality reduction method (Semi-Supervised Agglomerative Graph Embedding) In Section 3, we introduce an active learning strategy (Uncertainty Sampling), thereby providing the theoretical background for AdDReSS. and describe our method AdDReSS (Adaptive Dimensionality Reduction with Semi-Supervision). In Section 4, we outline the datasets, training parameters, and the performance measures used to evaluate the methodologies described in this work. In Section 5, we demonstrate the performance of the comparative methodologies on the basis of learning rate, classification accuracy, clustering performance, and variability, followed by concluding remarks in Section 6.

## 2 Review of Semi-Supervised Dimensionality Reduction Schemes

### 2.1 Notation

We denote a set E of samples *c*_*i*_, $c_{j}\in E$, *i*, *j* ∈ {1, 2, …, *N*}, where *N* is the number of samples in set E. Each sample *c*_*i*_ is represented by a 1 × *K* feature vector **x**_*i*_ ∈ *X*. We can formalize a dataset *X* as a *N* × *K* matrix containing *K* feature values for each of *N* samples. The goal of dimensionality reduction is to reduce the *N* × *K* matrix, defined by a 1 × *K* feature vector **x**_*i*_ ∈ *X*, where *k* < *K*, to a *N* × *k* matrix, where all samples *c*_*i*_ are defined by a 1 × *k* eigen-feature vector **y**_*i*_ ∈ *Y*. Label information may be introduced such that *ℓ*(*c*_*i*_) denotes the object class label of sample *c*_*i*_ as being a positive class (+1) or negative class (−1). Labels *ℓ*(*c*_*i*_) = 0 denotes that sample *c*_*i*_ is unlabeled.

### 2.2 Graph Embedding

NLDR methods, such as Graph Embedding [23], can be used to reduce samples *c*_*i*_ originally represented as *K*-dimensional vectors **x**_*i*_ ∈ *X* into *k*-dimensional vectors **y**_*i*_ ∈ *Y*, where *k* < *K*. To perform this transformation, data *X* is first represented as an affinity matrix *W*, which describes the similarity between all pairs of objects *c*_*i*_, *c*_*j*_ ∈ *S* as a graph *G* = {*V*, *E*}, where *V* represents all objects *c*_*i*_ and *c*_*j*_ as vertices, and *E* represents the edges which connect them.

Similarity is computed via the Gaussian diffusion kernel $\gamma =e^{-\frac{{\left|\right|x_{i}-x_{j}\left|\right|}_{2}}{\sigma }}$, which affects the weighting of the components in *W*. The kernel allows for a flexible local neighborhood constraint induced based on *σ*. A small *σ* narrows the size of the local neighborhood such that fewer points are deemed similar, whereas a large *σ* increases the size of the local neighborhood such that more points are similar. We set *σ* = max_*i*,*j*_||**x**_*i*_ − **x**_*j*_||_2_.

Alternatively, *E*, the edges in the graph *G*, expressed via the affinity matrix, *W*, can be pruned to further constrain local neighborhoods for NLDR. *E* can be defined based on a local neighborhood size determined by the number of nearest neighbors *κ*. For each *c*_*i*_, if *c*_*j*_ is one of the *κ*-nearest neighbors of *c*_*i*_, then we may include *c*_*j*_ in the set $K_{i}$ and we can express the edge as *E*(*c*_*i*_, *c*_*j*_) = 1. The weight matrix *W* represents a non-binary extension of the graph *G*, which takes into account the explicit similarity between objects *c*_*i*_ and *c*_*j*_ in terms of **x**_*i*_ and **x**_*j*_ such that

$$\begin{array}{c} W\left(x_{i},x_{j}\right)\;=\;\{\begin{array}{c} \gamma ,\;\text{if}\;c_{j}\in K_{i}\\ 0,\;\text{otherwise}. \end{array}, \end{array}$$ (1)

As performed in the normalized cuts algorithm [23], the affinity matrix is normalized such that

$$\begin{array}{c} \tilde{W}\left(x_{i},x_{j}\right)={\left(\sum_{ii}^{N}W(x_{ii},x_{j})\times \sum_{jj}^{N}W(x_{i},x_{jj})\right)}^{-1}W\left(x_{i},x_{j}\right). \end{array}$$ (2)

$\tilde{W}\left(x_{i},x_{j}\right)$ is used to solve the eigenvalue problem

$$\begin{array}{c} (D-\tilde{W})e=\lambda De, \end{array}$$ (3)

where *D* is a diagonal matrix containing the trace of $\tilde{W}$, and *e* are the eigenvectors. The embedding *Y*^*GE*^ is formed by taking the most dominant eigenvectors *e*_*β*_, *β* ∈ {1, 2, …, *k*}, corresponding to the *k* smallest eigenvalues *λ*_*β*_, where *k* corresponds to the dimensionality of *Y*^*GE*^.

### 2.3 Semi-Supervised Agglomerative Graph Embedding

Adding semi-supervised learning to DR is performed by modifying the Graph Embedding algorithm to introduce the label information *ℓ*(*c*_*i*_). A typical strategy for introducing label information into the Graph Embedding framework is to apply an additional set of weighting constraints to describe pairs of *c*_*i*_ and *c*_*j*_ with either the same (*ℓ*(*c*_*i*_) = *ℓ*(*c*_*j*_)) or different (*ℓ*(*c*_*i*_)≠*ℓ*(*c*_*j*_)) labels. We utilize a methodology used by Zhao et al. [35], SSAGE, which includes a multiplier to the Gaussian diffusion kernel $\gamma =e^{-\frac{{\left|\right|x_{i}-x_{j}\left|\right|}_{2}}{\sigma }}$, where *σ* = max_*i*,*j*_||**x**_*i*_ − **x**_*j*_||_2_, such that the affinity matrix is now defined as

$$\begin{array}{c} \hat{W}\left(x_{i},x_{j}\right)=\{\begin{array}{c} \gamma \left(1+\gamma \right),\;\text{if}\;\ell \left(c_{i}\right)=\ell \left(c_{j}\right)\;\text{and}\;c_{j}\in K_{i}\\ \gamma \left(1-\gamma \right),\;\text{if}\;\ell \left(c_{i}\right)\neq \ell \left(c_{j}\right)\;\text{and}\;c_{j}\in K_{i}\\ \gamma ,\;\text{if}\;\ell \left(c_{j}\right)=0\;\text{and}\;c_{j}\in K_{i}\\ 0,\;\text{otherwise} \end{array} \end{array}$$ (4)

$\hat{W}$ contains the weighted similarities between *c*_*i*_ and *c*_*j*_ based on (a) its position in *K*-dimensional space via the Gaussian diffusion kernel, (b) its proximity to its *κ* nearest neighbors, (c) whether that neighbor is of the same label class or not.

$\hat{W}$ is subsequently normalized via Eq (2) and the resulting normalized affinity matrix undergoes eigenvalue decomposition as performed in Eq (3). As with GE, the embedding *Y*^*SS*^ for SSAGE is formed by taking the most dominant eigenvectors *e*_*β*_, *β* ∈ {1, 2, …, *k*}, corresponding to the *k* smallest eigenvalues *λ*_*β*_, where *k* is the dimensionality of *Y*^*SS*^.

## 3 AdDReSS

### 3.1 Brief Overview

**Fig 1:**
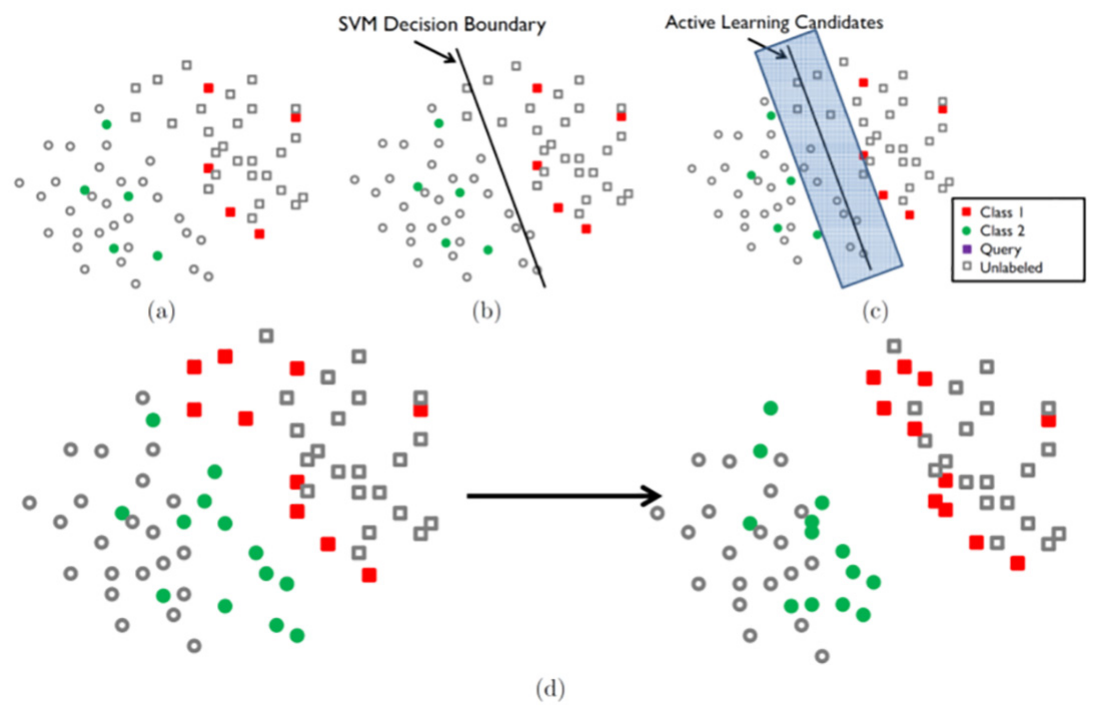
An example of how AdDReSS improves embedding by incorporating AL. (a) The original embedding representation generated by SSDR. (b) A support vector machine classifier is used as an active learner. (c) samples within the low dimensional embedding found to be difficult to classify are selected as candidates for training. (d) SSDR trained on the labels queried by AL provide greater separation of object classes in the low dimensional embedding.

The spirit of AdDReSS is embodied in Fig 1. Given an initial low-dimensional representation, a Support Vector Machine (SVM) [45] classifier is used to identify instances of the classes that are difficult to classify. The goal then, is to separate these two classes in a lower dimensional embedding representation such that each class is in a distinct region of the low dimensional embedding space. AdDReSS invokes AL to identify difficult to classify samples from within the embedding representation. These samples are subsequently used to train the semi-supervised agglomerative graph embedding (SSAGE) strategy to produce a more separable representation of the data. This process can be iterated to further refine the embedding representation.

### 3.2 Active Learning by Uncertainty Sampling for Identifying Ambiguous Samples

One can identify samples for AL by querying difficult to classify samples [5, 38, 40, 46, 47]. While many strategies have been investigated for AL using different classifiers, ultimately these differences were found not to be heavily correlated with classification performance [5]. For uncertainly sampling, a labeled set *S*_*tr*_ is first used to train a classifier. For each *S*_*tr*_, *γ* and *c* parameters are optimized by the grid search methodology proposed in Hsu et al. [48] and subsequently used to predict on the unlabeled set *S*_*ts*_. For each sample in the unlabeled set *S*_*ts*_, the classifier predicts the object class label *ℓ*(*c*_*i*_) with a certain probability that *c*_*i*_ belongs to that particular object class *ℓ*(*c*) (i.e. *P*(*ℓ*(*c*_*i*_) = 1)). We can define the most ambiguous samples as those with a probability of *P*(*ℓ*(*c*_*i*_)) = 0.5. We aim to find samples *c*_*i*_ nearest to *P*(*ℓ*(*c*_*i*_)) = 0.5 via the objective function

$$\begin{array}{c} \;\underset{c_{i}\in S_{ts}}{\mathrm{argmin}}\left|P(\ell \left(c_{i}\right)=1)-0.5\right|. \end{array}$$ (5)

These samples *c*_*i*_ are assigned to set *S*_*a*_. Labels *ℓ*(*c*_*i*_), *c*_*i*_ ∈ *S*_*a*_ are queried and these ambiguous samples are added to the training set

$$\begin{array}{c} \;S_{tr}=\left[S_{tr}\cup S_{a}\right]. \end{array}$$ (6)

Learning via the updated labels *ℓ*(*c*_*i*_), *c*_*i*_ ∈ *S*_*tr*_, we endeavor to improve classification performance compared to *S*_*tr*_ ∉ *S*_*a*_.

### 3.3 Algorithm

**Fig 2:**
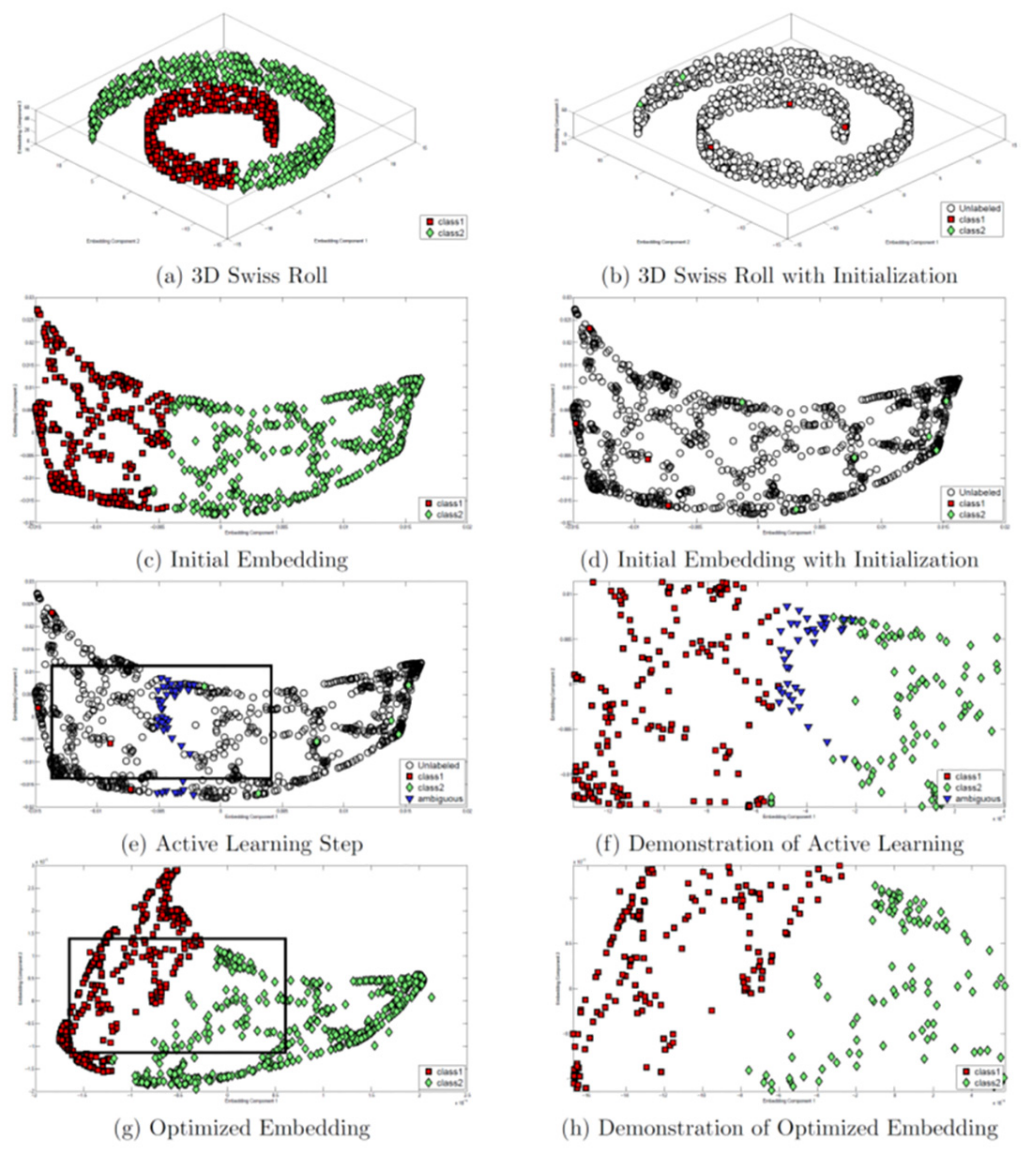
Swiss Roll example. (a) 3D Swiss Roll with all labels revealed. (b) 3D Swiss Roll with initial labels *ℓ*(*S*_*tr*_) revealed. (c) Initial 2D embedding with labels. (d) Initial 2D embedding with initial labels *ℓ*(*S*_*tr*_). (e) Ambiguous samples (in blue) are determined via active learning. (f) Region of the Swiss Roll at the class boundary (region is shown as a box in (e)). Note the selection of ambiguous samples (in blue) at the boundary between the two classes (in red and green). (g) Subsequent 2D embedding incorporating newly queried labels from the ambiguous samples. (h) Region near the class boundaries (shown as a box from (g)) revealing the increased separation between the two classes (in red and green) following application of the AdDReSS scheme.

The iterative Algorithm *AdDReSS* is presented below. Additionally, we employ the synthetic Swiss Roll example [24] presented in Fig 2 to guide the explanation of the AdDReSS algorithm. Fig 2(a) shows the 3-dimensional representation of the Swiss Roll dataset [24] shown with the two classes. The goal is to separate these two classes in a lower dimensional embedding representation such that each class is in a distinct region of the low dimensional embedding space. Fig 2 illustrates how the use of active learning is able to improve upon the separability of the two classes for this dataset.

Difficult to classify examples are identified by the SVM classifier in embedding space and are shown in blue in Fig 2(e). The newly identified objects discovered via AL attract towards similarly labeled samples already available to SSAGE and the classifier while repelling from dissimilarly labeled samples, thus creating the separation shown in Fig 2(g). Thus, it is clear that the discovery of difficult to classify labels can produce greater separation of the embedding as these samples are leveraged by SSAGE. The use of random sampling would probabilistically provide a uniform sampling of points in the dataset such that SSAGE could not leverage the samples at the classification boundary, resulting in a smaller degree of separation of object classes.

Line 0 of the algorithm refers to *Model Initialization*, the construction of the initial embedding *Y*^*Ad*^, and is illustrated in Fig 2(c) which shows the application of AdDReSS on the Swiss Roll dataset. The initialized embedding *Y*^*Ad*^ is created using data *X* via GE. In Fig 2(d), the revealed labels used for active learning are mapped onto *Y*^*Ad*^.

The subsequent illustrations, Fig 2(e) and 2(g), represent successive runs of *Active Learning* and *Model Refinement* via SSDR, respectively, which are contained within the while loop of the algorithm (lines 2-7).

**Algorithm** *AdDReSS*

**Input**: *X*, *ℓ*(*S*_*tr*_)

**Output**: *Y*^*Ad*^

begin

  0. Build initial embedding *Y*^*Ad*^ using *X*, *ℓ*(*c*) = { } via Eq (3)

  1. **while** *S*_*ts*_ ≠ { }

  2.   Train classifier using *Y*^*Ad*^, *ℓ*(*S*_*tr*_)

  3.   Predict *ℓ*(*c*_*i*_) in *S*_*ts*_ using classifier model in Step 2

  4.   Identify ambiguous samples from *c*_*i*_ ∈ *S*_*ts*_ via Eq (5)

  5.   Query labels *ℓ*(*c*_*i*_), *c*_*i*_ ∈ *S*_*a*_

  6.   Update *S*_*tr*_ via Eq (6)

  7.   Update embedding *Y*^*Ad*^ using updated *ℓ*(*S*_*tr*_) via Eq (4)

  8. **end**

  9. *return* *Y*^*Ad*^

end

Lines 2-6 of the algorithm represent the *Active Learning* component described earlier in Section 3.2, where ambiguous samples are identified based on the results of a trained classifier. Although Doyle et al. [5] have suggested that the particular choice of active learner is not significantly correlated with classifier performance, we have chosen the Support Vector Machine (SVM) classifier to identify the ambiguous samples for the following reasons. Firstly, SVMs have been shown to be highly generalizable to new unseen testing data, suggesting that the algorithm can consistently identify ambiguous samples [45, 46]. Secondly, SVMs have been heavily investigated and employed for active learning [49, 50]. Finally, SVMs, like GE, operate on a kernel representation of the data, allowing for seamless identification of ambiguous samples derived from the kernel space in construction of the embeddings. A linear kernel was used based on the assumption that the NLDR method GE provides a linearly separable embedding as GE is able to account for non-linear data. We have previously shown the ability of linear kernel SVM to separate biomedical data using low dimensional representations from NLDR methods [13].

Fig 2(e) shows a visualization of the ambiguous samples found via SVM classification of Fig 2(d). Difficult to classify samples (shown as blue points) are found at the intersection of the two labeled classes (Fig 2(f)). New labels are obtained for these samples and added to the training set, completing the active learning phase (lines 2-6).

Line 7 of the algorithm represents the *Model Refinement* component where the updated label set *ℓ*(*S*_*tr*_) found via active learning is used to create an improved embedding representation via SSDR (Fig 2(g)). This representation demonstrates an improvement upon the previous embedding (Fig 2(c)). These steps of identifying samples (Fig 2(e)) and generating an optimized representation (Fig 2(g)) may be repeated until there are no additional unlabeled samples available for querying or until there is a lack of ambiguous samples to be queried.

## 4 Experimental Design

### 4.1 Dataset Description

A total of 4 datasets ($D_{1}$—$D_{4}$) were used in this study. These datasets include: $D_{1}$: gene-expression of prostate cancer, $D_{2}$: protein expression of ovarian cancer, $D_{3}$: breast histology image data, and $D_{4}$: synthetic brain image data. The datasets are summarized in Table 1.

**Table 1:** Datasets used for evaluation.

*BiomedicalDatasets*	*Description*	*Features*
$D_{1}$: Prostate Cancer	52 Tumor, 50 Normal	Gene Expression (12600)
$D_{2}$: Ovarian Cancer	162 Tumor, 91 Normal	Protein Expression (15154)
$D_{3}$: Breast Histopathology	316 Mitotic nuclei, 8592 Non-mitotic nuclei	Multi-window RGB Intensities (758)
$D_{4}$: BrainWeb	5,975 total Grey Matter and White Matter pixels	Texture (14)
109 × 131 image	2607 Grey Matter, 3368 White Matter	

#### 4.1.1 $D_{1}$: Gene Expression of Prostate Cancer

*Preprocessing*: Gene expression data [8] was acquired from the Biomedical Kent-Ridge Repositories (http://datam.i2r.a-star.edu.sg/datasets/krbd/), consisting of high quality expression profiles from 52 prostate tumors and 50 non-tumor (normal) prostate samples. The samples are derived from oligonuleotide microarrays containing probes for 12,600 genes.

*Feature Extraction*: No additional feature extraction was performed and all embeddings were calculated directly from the provided data. For all results, the *K* = 12,600 dimensional dataset was reduced down to dimensionality *k* ∈ {2,3}.

#### 4.1.2 $D_{2}$: Protein Expression of Ovarian Cancer

*Preprocessing*: The study [4], obtained from the Biomedical Kent-Ridge Repositories (http://datam.i2r.a-star.edu.sg/datasets/krbd/) uses proteomic spectra extracted from serum to distinguish 91 neoplastic from 162 non-neoplastic disease within the ovary. The proteomic spectra generated by SELDI mass spectroscopy for each sample contains the relative amplitude of 15,154 intensities at each molecular mass / charge (M/Z) identity.

*Feature Extraction*: No additional feature extraction was performed and all embeddings were calculated directly from the provided data. For all results, the *K* = 15,154 dimensional protein spectra was reduced down to dimensionality *k* ∈ {2,3}.

#### 4.1.3 $D_{3}$: Mitotic Detection in Breast Cancer Histological Images

*Preprocessing*: This dataset was obtained from the mitosis 2012 ICPR contest [43]. The task is mitotic nuclei identification (http://www.ipal.cnrs.fr/event/icpr-2012). Five breast cancer biopsy slides are stained with hematoxylin and eosin (H&E). In each slide, pathologists selected 10 high power fields (HPF) at 40X magnification. An HPF has a size of 512 × 512 *μ*m^2^. Each HPF was scanned by an Aperio XT scanner at 0.2456 *μ*m per pixel to create a 2084 × 2084 image. These 50 HPFs contain 316 annotated mitotic nuclei in total and an automated nuclear detection algorithm is used to select an additional 8592 non-mitotic nuclei for a total of 8908 nuclei.

**Fig 3:**
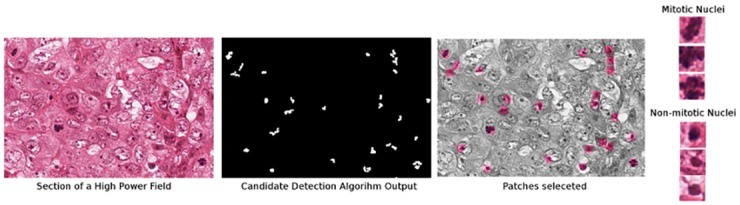
Selection of mitotic and non-mitotic nuclei from the MITOS2012 dataset. A nuclei candidate detection algorithm is used and patches centered at each candidate centroid are extracted.

The automated nuclei detection algorithm involves the application of a blue ratio transformation [51] upon each HPF followed by a global thresholding via Otsu’s method [52] to obtain a binary image. Following a morphologic opening operation applied to the binary image, we assign the centroid of each connected component as a nucleus. Patches containing each nucleus as its centroid are illustrated in Fig 3.

*Feature Extraction:* 8908 nuclei are processed using the centroid of each nuclei as the center of an 8 × 8 image. In this manner, 8 × 8 images are generated for 4 resolutions (20X, 10X, 5X, and 2.5X). These RGB intensities for all pixels across all the 4 patch resolutions are subsequently vectorized such that 8 × 8 × 4 × 3 = 768 RGB intensities [53]. The *K* = 768 dimensional feature vector was reduced down to dimensionality *k* ∈ {2, 3}.

#### 4.1.4 $D_{4}$: BrainWeb Images

*Preprocessing*: Synthetic brain data [44] was acquired from the Montreal Neurological Institute (http://www.bic.mni.mcgill.ca/brainweb/), consisting of simulated proton density (PD) MRI brain volumes at various noise and bias field inhomogeneity levels. Gaussian noise artifacts have been added to each pixel in the image, while inhomogeneity artifacts were added via pixel-wise multiplication of the image with an intensity non-uniformity field. Parameters for Gaussian noise artifacts (NO) ranged from 1% to 9% noise. Similarly, intensity non-uniformity (RF) ranged from 0 to 40%. Images were acquired at a slice thickness of 1mm. The dataset provides corresponding labels for each of the separate regions within the brain, including white matter (WM) and grey matter (GM). A single slice is used in this study comprising WM and GM alone (ignoring other brain tissue classes).

*Feature Extraction*: 14 texture features [54] were extracted from each image on a per-pixel basis: angular second moment, contrast, correlation, sum of squares variance, inverse difference moment, sum average, sum variance, sum entropy, entropy, difference variance, difference entropy, two features of information measure of correlation, and max correlation coefficient. These features are based on calculating statistics from a gray level intensity co-occurrence matrix constructed from the image, and were chosen due to previously demonstrated discriminability between cancerous and non-cancerous regions in the prostate [55] and different types of brain matter [56] for MRI data. For all results, the *K* = 14 dimensional texture feature space is reduced to dimensionality *k* ∈ {2, 3}.

### 4.2 Comparative Strategies

Our experimental design was constructed to highlight the differences between embeddings generated via three schemes: (1) Graph Embedding (GE), (2) Semi-Supervised Agglomerative Graph Embedding (SSAGE) and (3) AdDReSS, a SSDR method using active learning. A summary of the methods is presented in Table 2. An empirical maximum (“Empirical Max”) is also shown in some of the plots to demonstrate a ceiling for classification performance. The empirical maximum is calculated as the highest *ϕ*^*Acc*^ obtained for any single iteration of *Y* such that $\phi^{EM}=\max_{i,l}[\phi_{i}^{Acc}\left(Y_{l}^{Ac}\right)]$, where *i* ∈ {1, 2, …, *n*} denotes specific run of *Y*^*Ac*^ with a unique initial training set *S*_*ts*_.

**Table 2:** Strategies compared in this work.

*ReductionStrategy*	*Description*	*KeyEquation*
*GE* [23]	Unsupervised NLDR method which does not use any label information	$W\left(x_{i},x_{j}\right)\;=\;\{\begin{array}{c} \gamma ,\;\text{if}\;c_{j}\in K_{i}\\ 0,\;\text{otherwise} \end{array}$
*SSAGE* [35]	SSDR method which utilizes random sampling	$\hat{W}\left(x_{i},x_{j}\right)\;=\;\{\begin{array}{c} \gamma \left(1+\gamma \right),\;\text{if}\;\ell \left(c_{i}\right)=\ell \left(c_{j}\right)\;\text{and}\;c_{j}\in K_{i}\\ \gamma \left(1-\gamma \right),\;\text{if}\;\ell \left(c_{i}\right)\neq \ell \left(c_{j}\right)\;\text{and}\;c_{j}\in K_{i}\\ \gamma ,\;\text{if}\;\ell \left(c_{j}\right)=0\;\text{and}\;c_{j}\in K_{i}\\ 0,\;\text{otherwise} \end{array}$
*AdDReSS*	SSDR method using active learning	$\hat{W}\left(x_{i},x_{j}\right)\;=\;\{\begin{array}{c} \gamma \left(1+\gamma \right),\;\text{if}\;\ell \left(c_{i}\right)=\ell \left(c_{j}\right)\;\text{and}\;c_{j}\in K_{i}\\ \gamma \left(1-\gamma \right),\;\text{if}\;\ell \left(c_{i}\right)\neq \ell \left(c_{j}\right)\;\text{and}\;c_{j}\in K_{i}\\ \gamma ,\;\text{if}\;\ell \left(c_{j}\right)=0\;\text{and}\;c_{j}\in K_{i}\\ 0,\;\text{otherwise} \end{array}$
		$\underset{c_{i}\in S_{ts}}{\mathrm{argmin}}\left|P(\ell \left(c_{i}\right)=1)-0.5\right|.$

### 4.3 Embedding Parameters

Embeddings *Y*^*Ad*^ and *Y*^*SS*^ for AdDReSS and SSAGE, respectively, (refer to Sections 2.3 and 3.3 for more details) were generated with 20 different randomly selected training sets *S*_*tr*_ of training samples. Measures designed to evaluate each embedding were calculated across multiple iterations, $Y_{l\%}^{Ad}$, corresponding to an embedding for a percentage *l* of revealed labels *ℓ*(*c*_*i*_). These trials were repeated across a range of parameters for each dataset $D_{1}$-$D_{3}$ (as described in Section 4). Embeddings *Y*^*GE*^ were also generated for unsupervised GE (refer to Section 2.2 for more details) for comparison, but since no label information is used, only one embedding is obtained across all label iterations for each parameter set. Optimal *κ* parameters *κ* ∈ {2, …, *n* − 1} were selected for all experiments, where *n* is the number of samples in the dataset.

### 4.4 Training Parameters

Each dataset is divided equally into training and testing pools, $E_{tr}$ and $E_{ts}$, respectively, for the purpose of an unbiased evaluation of the resulting *Y*. Random stratified sampling was performed such that samples for each of $E_{tr}$ and $E_{ts}$ are randomly chosen such that the number of positive and negative class labels *ℓ*(*c*) is the same in both $E_{tr}$ and $E_{ts}$. Note that $E_{tr}$ and $E_{ts}$ are distinct from the training and testing sets *S*_*tr*_ and *S*_*ts*_ used for querying samples for active learning. *S*_*tr*_ and *S*_*ts*_ are solely used for construction of the embedding and make up the entirety of the training pool $E_{tr}$, described in this section such that $E_{tr}$ = [*S*_*tr*_ ∪ *S*_*ts*_]. Meanwhile, the labels $\ell (E_{ts})$ in the testing pool are used only for analysis and are not used for constructing *Y*.

### 4.5 Performance Evaluation

We evaluate AdDReSS on the basis of summarize 7 evaluation measures summarized in Table 3. 5 measures have been previously explored, and we refer the reader to the provided citations and Appendix for additional details on Random Forest classification accuracy [57], Silhouette Index [58], and Raghavan Efficiency [59]. Additionally, we present 2 new additional measures (Maximum Query Efficiency and Maximum Information Gain) to illustrate the learning rates provided via an active learning approach as compared to a random sampling approach. These two measures are described below.

**Table 3:** Summary of Evaluation Measures.

*EvaluationMeasure*	*Description*
Classification Accuracy (*ϕ*^*Acc*^) [57]	Classifier accuracy (Acc) is calculated to evaluate class separability within the embedding
Silhouette Index (*ϕ*^*SI*^) [58]	Silhouette Index (SI) offers an independent measure to quantify the separation of multiple classes in the embedding. SI can detect more subtle changes in the embedding with regards to overall class separation compared to classification accuracy.
Embedding Variance via Classification Accuracy (*ρ*^*Acc*^) [57]	It is anticipated that active learning will be able to consistently identify training instances, *S*_*a*_, which will lead to improved classification, whereas random sampling will show more varied improvement due to the variance in the specific training instances chosen.
Embedding Variance via Silhouette Index (*ρ*^*SI*^) [58]	Similar to *ρ*^*Acc*^, we also aim to quantify the variance of the embedding with regards to the Silhouette Index, which reflects the separability of the two object classes in terms of the Euclidean distance between data points in the embedding *Y*.
Raghavan Efficiency (*ϕ*^*Eff*^) [59]	Raghavan Efficiency describes the rate of learning among active learning algorithms. We use *ϕ*^*Eff*^ to compare the overall learning rate between 1) AdDReSS vs GE, 2) SSAGE vs GE and 3) AdDReSS vs SSAGE.
Maximum Query Efficiency (*ϕ*^*MQE*^)	Maximum Query Efficiency is the ratio between the maximum difference in the number of labels necessary to achieve the same classification performance and the number of potential queries.
Maximum Information Gain (*ϕ*^*MIG*^)	We define maximum information gain as the maximum difference in classification performance *ϕ*^*Acc*^ at a given label query amount *l*

#### 4.5.1 Evaluation of Maximum Query Efficiency (*ϕ*^*MQE*^)

While Raghavan Efficiency is useful as an overall measure, there remain important insights that cannot be surmised by the global measure. One example is the cost savings associated with using active learning based dimensionality reduction compared to with traditional SSDR using random sampling. Maximum Query Efficiency is the ratio between the maximum difference in the number of labels necessary to achieve the same classification performance and the number of potential queries such that

$$\begin{array}{c} \phi^{MQE}=\max_{\phi^{Acc}}[\frac{l^{SS}-l^{Ad}}{N}], \end{array}$$ (7)

where *l*^*SS*^ and *l*^*Ad*^ refer to the mean number of labels queried by SSAGE and AdDReSS, respectively, to achieve a classification performance *ϕ*^*Acc*^. *N* refers to the number of total samples $c_{i}\in E$. A larger *ϕ*^*MQE*^ is indicative of greater savings in terms of labels queried.

#### 4.5.2 Evaluation of Maximum Information Gain (*ϕ*^*MIG*^)

Another useful measure of active learning performance is the maximum information gain from using a particular algorithm of choice. We define maximum information gain as the maximum difference in classification performance *ϕ*^*Acc*^ at a given label query amount *l*, such that

$$\begin{array}{c} \phi^{MIG}=\max_{l}[\bar{\phi^{Acc}\left(Y_{l}^{Ad}\right)}-\bar{\phi^{Acc}\left(Y_{l}^{SS}\right)}]. \end{array}$$ (8)

A larger *ϕ*^*MIG*^ refers to a larger difference between the classification performance between embeddings constructed by AdDReSS and embeddings generated by SSAGE.

## 5 Results and Discussion

### 5.1 Evaluation via Classifier Accuracy (*ϕ*^*Acc*^)

**Fig 4:**
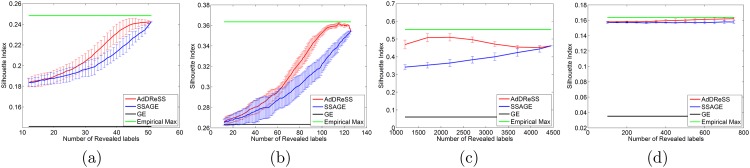
Evaluation of Classification Accuracy. Number of instances for which labels were revealed versus mean *ϕ*^*Acc*^ for AdDReSS, SSAGE, GE, and the maximum empirically derived *ϕ*^*Acc*^ across all runs is shown for (a) $D_{1}$, (b) $D_{2}$, (c) $D_{3}$ and (d) $D_{4}$. Standard deviation of *ϕ*^*Acc*^ shown as error bounds at each *l*.

Fig 4 shows the classification performance of AdDReSS against SSAGE and GE on four biomedical datasets ($D_{1}$—$D_{4}$), where different amounts of labeled data *l* are revealed to the classifier. We notice greater *ϕ*^*Acc*^ for AdDReSS across all amounts of revealed labels *l*. The accuracy curve corresponding to AdDReSS also approaches the empirical maximum *ϕ*^*Acc*^ at a faster rate compared to SSAGE. GE is also shown for each case as a comparison. The use of sufficient labeled instances suggests a clear advantage in employing semi-supervision for DR. Furthermore, the improved performance of AdDReSS over SSAGE across all labeled instances reveals a measurable difference in *ϕ*^*Acc*^ at a point between the minimum *l* = 10% and the maximum number of revealed labels *l* = 50%. This is due to the fact that for small training size, *l* = 10%, there is a significant overlap in *S*_*tr*_ for AdDReSS and SSAGE due to the identical initialization *S*_*tr*_. Similarly at *l* = 50%, training samples are exhausted from the pool $E_{tr}$, such that $S_{tr}=E_{tr}$ for both AdDReSS and SSAGE. Therefore, the greatest measurable difference between $\phi^{Acc}\left(Y_{l}^{Ad}\right)$ and $\phi^{Acc}\left(Y_{l}^{SS}\right)$ can be seen where 10%<*l* < 50%, reflecting the difference in the active learning and random sampling strategies towards the composition of *c*_*i*_ ∈ *S*_*tr*_, and subsequently, towards the resulting embeddings $Y_{l}^{Ad}$ and $Y_{l}^{SS}$.

### 5.2 Evaluation via Silhouette Index (*ϕ*^*SI*^)

**Fig 5:**
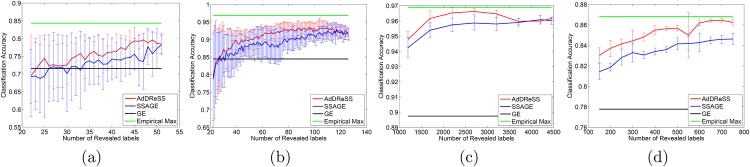
Evaluation of Silhouette Index. Number of instances for which labels were revealed versus mean *ϕ*^*SI*^ for AdDReSS, SSAGE, GE, and the maximum empirically derived *ϕ*^*SI*^ across all runs is shown for (a) $D_{1}$, (b) $D_{2}$, (c) $D_{3}$ and (d) $D_{4}$. Standard deviation in *ϕ*^*SI*^ shown as error bounds at each *l*.

In Fig 5, we compared AdDReSS against SSAGE and GE in terms of *ϕ*^*SI*^ on datasets ($D_{1}$—$D_{4}$) by revealing different amounts of labeled data *l*. Compared to *ϕ*^*Acc*^, there appears to be greater separation for *ϕ*^*SI*^ between the semi-supervised methods compared to GE. This in turn seems to suggest that the separation of the object classes in the embedding space is more pronounced. Furthermore, *ϕ*^*SI*^(*Y*^*Ad*^) outperforms *ϕ*^*SI*^(*Y*^*SS*^) across all *l*. In contrast to *ϕ*^*Acc*^, the improvement in *ϕ*^*SI*^ tends to continue with increasing numbers of revealed labeled information *l*. Only when the revealed labeled information is nearly *l* = 50% does *ϕ*^*SI*^ approach its empirical maximum *ϕ*^*SI*^.

### 5.3 Evaluation of Variance (*ρ*^*Acc*^, *ρ*^*SI*^)

**Fig 6:**
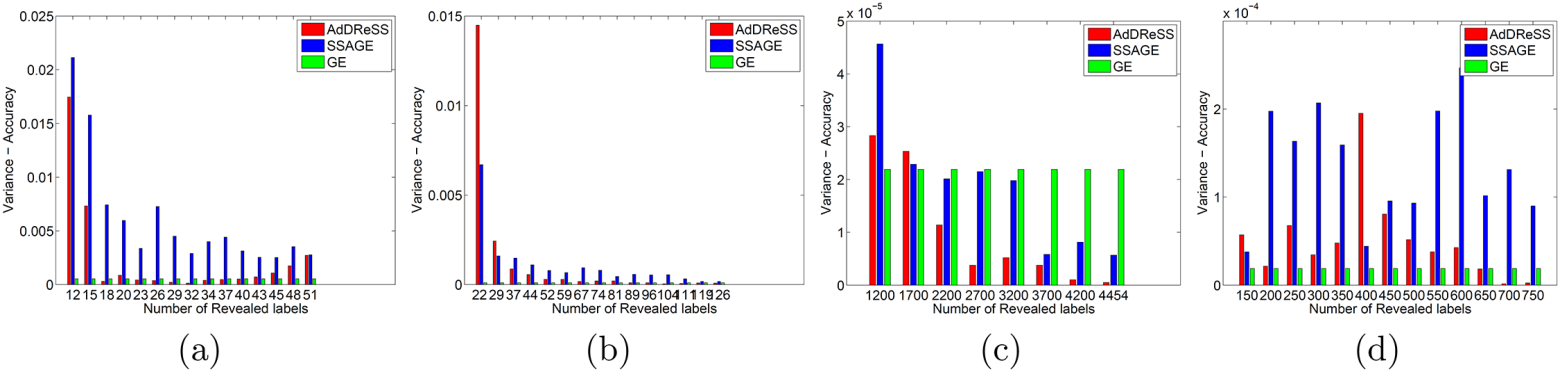
Evaluation of Variance for Classification Accuracy. Variance of *ϕ*^*Acc*^ at selected numbers of instances for which labels were revealed for AdDReSS, SSAGE, GE are shown for (a) $D_{1}$, (b) $D_{2}$, (c) $D_{3}$, and (d) $D_{4}$.

In Fig 6, we compare variance *ϕ*^*Acc*^ across varied amounts of revealed labels *l* for *Y*^*Ad*^, *Y*^*SS*^ and *Y*^*GE*^. In $D_{4}$, we notice very small differences in *ϕ*^*Acc*^, as *ρ*^*Acc*^ is found to be on average less than 0.0003 for all values of *l*. Nevertheless, we can view significant differences between *ρ*^*Acc*^ of AdDReSS and SSAGE, with AdDReSS showing *ρ*^*Acc*^ < 0.0001 in all but one instance, and most instances of SSAGE showing *ρ*^*Acc*^ > 0.0001. We notice greater differences in *ρ*^*Acc*^ for $D_{1}$ and $D_{2}$ in Fig 6(a) and 6(b) respectively, as both AdDReSS and SSAGE are more sensitive to the composition of initial training *c*_*i*_ ∈ *S*_*tr*_, reflected in the higher *ρ*^*Acc*^ when *l* < 10%. *ρ*^*Acc*^ is subsequently seen to decrease with increasing *l* as more training samples are queried by the active learner. For all experiments in $D_{1}$, AdDReSS shows more consistency in *ϕ*^*Acc*^ as demonstrated by lower *ρ*^*Acc*^ compared to SSAGE. Furthermore, AdDReSS shows similar *ρ*^*Acc*^ values when compared to the unsupervised GE method, which is reflective of the precision of the classifier. The same trends can be seen in $D_{2}$ for *l* > 28% (Fig 6(b)), where over 29 revealed labeled instances were used and AdDReSS shows lower *ρ*^*Acc*^ compared to SSAGE. Similar to $D_{4}$, there are very small differences in *ϕ*^*Acc*^ (less than 0.00005) across all *l*. However, AdDReSS shown to have a lower *ϕ*^*Acc*^ than SSAGE in all but 1 case, where the difference between AdDReSS and SSAGE is extremely small.

**Fig 7:**
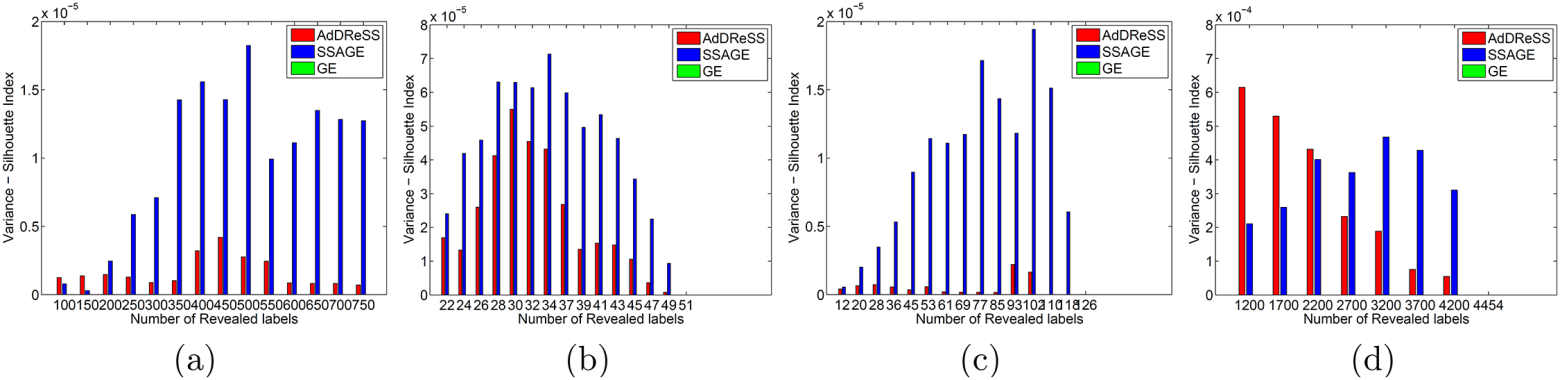
Evaluation of Variance for Silhouette Index. Variance of *ϕ*^*SI*^ at selected numbers of instances for which labels were revealed for AdDReSS, SSAGE, GE are shown for (a) $D_{1}$, (b) $D_{2}$, (c) $D_{3}$, and (d) $D_{4}$. GE shows zero variance as labeled information does not affect the embedding for GE.

In Fig 7, we demonstrate more consistent embeddings *Y*^*Ad*^ compared to *Y*^*SS*^ as demonstrated by a lower *ρ*^*SI*^. However, unlike with *ρ*^*Acc*^, *ρ*^*SI*^ tends to increase with increasing *l*. In all three of four datasets $D_{1}$-$D_{4}$, we notice SSAGE to have greater *ρ*^*SI*^ than AdDReSS and up to 3 or 4 times greater for $D_{2}$ and $D_{4}$. In the final dataset $D_{3}$, *ρ*^*SI*^ of AdDReSS steadily decreases with increasing *l*, whereas *ρ*^*SI*^ of SSAGE experiences a slight increase with ascending *l*. These trends in Figs 5 and 7 are reflective of the ability of the embedding to converge more quickly with increasing *l* for AdDReSS compared to SSAGE. The embedding for GE does not change with respect to *l*, therefore, there is no change in *ϕ*^*SI*^, and *ρ*^*SI*^ = 0 in any of the cases. These results are suggestive of a embedding representation *Y*^*Ad*^ which is more stable than *Y*^*SS*^, and is robust to the specific *c*_*i*_ ∈ *S*_*tr*_ used to initialize AdDReSS.

### 5.4 Evaluation via Raghavan Efficiency (*ϕ*^*Eff*^)

**Fig 8:**
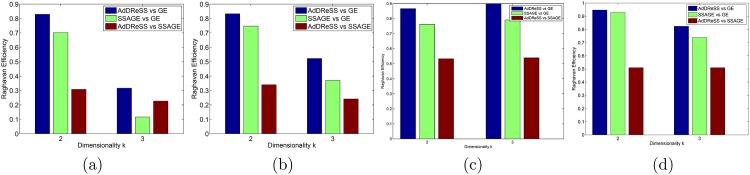
Evaluation of Raghavan Efficiency. *ϕ*^*Eff*^ for *k* ∈ {2, 3} shows the comparative efficiency between AdDReSS and GE, SSAGE and GE, and AdDReSS and SSAGE for (a) $D_{1}$, (b) $D_{2}$, (c) $D_{3}$, and (d) $D_{4}$.

In Fig 8, we show the overall differences in efficiency between each pair of methods (1) AdDReSS vs SSAGE, 2) AdDReSS vs GE, and 3) SSAGE vs GE) employed for this study via *ϕ*^*Eff*^. In all cases, AdDReSS outperforms SSAGE in terms of *ϕ*^*Eff*^. Furthermore, the large positive *ϕ*^*Eff*^(*Y*^*Ad*^|*Y*^*SS*^) values are consistent to what is seen in Fig 4, where AdDReSS shows greater *ϕ*^*Acc*^ for varying proportions of revealed labels *l*.

In investigating dimensionality, *ϕ*^*Eff*^(*Y*^*Ad*^|*Y*^*SS*^) is slightly higher overall when *k* = 2 for $D_{1}$ and $D_{2}$ compared to when *k* = 3, but show similar *ϕ*^*Eff*^(*Y*^*Ad*^|*Y*^*SS*^) for the imaging datasets $D_{3}$ and $D_{4}$. While the imaging datasets do not show much of a difference in *ϕ*^*Eff*^(*Y*^*Ad*^|*Y*^*SS*^) between dimensionalities, AdDReSS appears to show a pronounced difference in efficiency with fewer dimensions when evaluating the gene and protein expression datasets. While it is unclear why this difference is pronounced in the gene expression and proteomic datasets, overall, the results suggest that utilizing active learning could be used to represent the data with fewer features compared to random sampling.

The improvement in efficiency afforded by AdDReSS compared to SSAGE is summarized in Table 4 using GE as the baseline. Table 4 shows the percentage increase between *ϕ*^*Eff*^(*Y*^*Ad*^|*Y*^*GE*^) and *ϕ*^*Eff*^(*Y*^*SS*^|*Y*^*GE*^) for all datasets $D_{1}$-$D_{4}$. Overall, the mean percentage improvement in *ϕ*^*Eff*^ across all datasets was found to be +10.52% for *k* = 2 and +60.05% for *k* = 3 from using AdDReSS instead of SSAGE, suggesting that AdDReSS appears to outperform SSAGE as the number of dimensions begins to increase.

**Table 4:** Percent improvement in Raghavan efficiency via AdDReSS over SSAGE.

	$D_{1}$	$D_{2}$	$D_{3}$	$D_{4}$	Mean
*k* = 2	+18.09%	+11.53%	+15.79%	+1.94%	+11.84%
*k* = 3	+172.41%	+40.95%	+15.38%	+11.49%	+60.05%
Mean	+95.25%	+26.24%	+15.59%	+6.71%	+35.95%

### 5.5 Evaluation via Maximum Information Gain (*ϕ*^*MIG*^)

**Fig 9:**
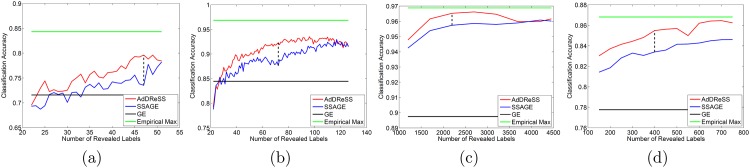
Evaluation of Maximum Information Gain. *ϕ*^*MIG*^ shows areas of maximum information gain (shown as a dashed black line) in terms of the difference in *ϕ*^*Acc*^ between AdDReSS and SSAGE for (a) $D_{1}$, (b) $D_{2}$, (c) $D_{3}$, and (d) $D_{4}$.

In Fig 9, we show the maximum amount of information gain that can be achieved via AdDReSS compared to SSAGE for each dataset. For $D_{4}$, *ϕ*^*MIG*^ = 0.0208, which means there is a maximum improvement in *ϕ*^*Acc*^ of over 2% (from 0.8340 to 0.8548) due to AdDReSS compared to SSAGE. This improvement in *ϕ*^*Acc*^ via *Y*^*Ad*^ is equivalent to 60 additional correctly classified samples for $D_{4}$ compared to *Y*^*SS*^. In Fig 9(a), when *l* = 46% (47 labels revealed), $D_{1}$ shows *ϕ*^*MIG*^ = 0.0608, with over an 8% improvement in *ϕ*^*Acc*^ when using AdDReSS compared to SSAGE. For $D_{2}$, *ϕ*^*MIG*^ = 0.0465, with an improvement from 0.8764 to 0.9228 in terms of *ϕ*^*Acc*^ and the best improvement is found when *l* < 30% (less than 72 labels revealed). Lastly, for $D_{3}$, *ϕ*^*MIG*^ = 0.0079, which is significant given the high overall classification performance in the dataset. The maximum information gain also occurs when *l* < 30% for $D_{3}$. The results for *ϕ*^*MIG*^ suggest a faster rate of learning when using AdDReSS compared to SSAGE.

### 5.6 Evaluation via Maximum Query Efficiency (*ϕ*^*MQE*^)

**Fig 10:**
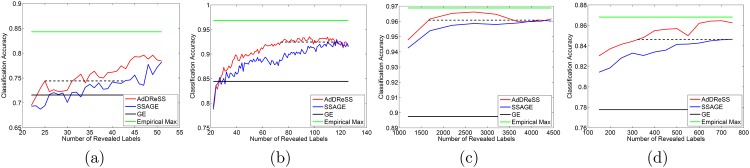
Evaluation of Maximum Query Efficiency. *ϕ*^*MQE*^ describes the maximum efficiency in terms of queried labels given the same *ϕ*^*Acc*^ (shown as a dashed black line) between AdDReSS and SSAGE for (a) $D_{1}$, (b) $D_{2}$, (c) $D_{3}$, and (d) $D_{4}$.

Fig 10 illustrates the number of fewer labels required for AdDReSS to achieve the same classification performance *ϕ*^*Acc*^ as SSAGE. For $D_{4}$, *ϕ*^*MQE*^ = 0.0698, which reflects the fact that AdDReSS requires an average of 417 fewer labels than SSAGE to achieve *ϕ*^*Acc*^ = 0.8462. Stated another way, SSAGE required the use of an additional 6.98% of the labels $l\left(c_{i}\right),c_{i}\in E$, to achieve the same performance as AdDReSS. For $D_{1}$, *ϕ*^*MQE*^ = 0.1748. While an average of 25 revealed labeled instances were used to achieve *ϕ*^*Acc*^ = 0.74 for AdDReSS, SSAGE required an average of 43 revealed labeled instances in order to achieve the same *ϕ*^*Acc*^. Similarly, for $D_{2}$, *ϕ*^*MQE*^ = 0.1730, such that AdDReSS required, on average, 74 labels to achieve *ϕ*^*Acc*^ = 0.9244 while SSAGE required nearly the entire training pool, $E_{tr}$, of 126 labels, as shown in Fig 10(c). Although $D_{3}$ showed a relatively small *ϕ*^*MIG*^, the *ϕ*^*MQE*^ = 0.2817, which results in 2509 fewer training cases for *Y*^*Ad*^ to achieve the same classification accuracy of *Y*^*SS*^ at *l* = 4178. Put another way, *Y*^*SS*^ required 2.503 times as many training samples to achieve the classification performance of *Y*^*Ad*^ at *l* = 1669. Overall, for $D_{1}-D_{4}$, AdDReSS was able to achieve the same classification accuracy as SSAGE while utilizing a mean of 48.8% (and up to 60%) fewer labels.

## 6 Concluding Remarks

In this work, we presented a novel nonlinear dimensionality reduction methodology, Adaptive Dimensionality Reduction with Semi-Supervision (AdDReSS), which attempts to seamlessly integrate active learning into semi-supervised dimensionality reduction (SSDR) to yield low dimensional data representations of high dimensional data. To date, no methods that we are aware of, have demonstrated the utility of active learning for improving low dimensional data representations. These representations yield greater classification accuracy and class separability while using fewer class labels. AdDReSS attempts to address the problems of classifying ‘big data’ and the very real problem of often not having class labels or annotations with which to train a classifier. Our scheme employs the use of active learning to query fewer labels which contribute the most towards building low dimensional embeddings with high object class separability and classification performance. We quantified the differences between AdDReSS and SSAGE on problems involving imaging and non-imaging channels from 4 distinct biomedical datasets (MR brain imaging, prostate gene expression, ovarian proteomic spectra, and breast histology images). Based on the results assessed over 8000 experiments, we make the following observations:

- AdDReSS has a greater predictive potential compared to SSAGE and GE based on classification accuracy when different numbers of instances have their labels revealed.
- AdDReSS achieved a higher Silhouette Index compared to SSAGE and GE, suggestive of an embedding with greater separation between the object classes.
- In comparisons of overall efficiency, AdDReSS learns at a faster rate of convergence to the maximum possible accuracy compared to SSAGE and GE, measured by a mean 35.95% increase in Ragahavan efficiency.
- The potential savings in terms of the number of labels to be queried to achieve the same classification accuracy was shown to be up to 56% for AdDReSS compared to SSAGE across the datasets considered.
- AdDReSS was also found to be more robust to randomized training set initialization, in that it appeared to have a lower variance in terms of classification accuracy and Silhouette Index compared to SSAGE in the datasets considered.

Our findings suggest that active learning has a measurable effect compared to random sampling on SSAGE for embedding construction and that AdDReSS could be a powerful data analysis and classification tool for high dimensional biomedical data, especially in scenarios where partial or incomplete annotations and class labels are available. Future work will involve further evaluation of the effects of AL on SSDR methods beyond the ones considered in this paper.

## Appendix

### Evaluation of Classification Accuracy (*ϕ*^*Acc*^)

Classifier accuracy (Acc) is calculated to evaluate class separability within the embedding.

$$\begin{array}{c} \phi^{Acc}=\frac{TP+TN}{TP+TN+FP+FN}. \end{array}$$ (9)

where TP is the number of true positives, TN is the number of true negatives, FP is the number of false positives, and FN is the number of false negatives.

Specifically, a Random Forest classifier (or bagged decision tree classifiers) [57] has been used due to its robustness and to reduce bias by selecting a different classifier than the one used for query ambiguous samples (in our case, an SVM classifier). The Random Forest classifier is constructed using 50 decision tree classifiers each trained on a random third of the training pool $E_{tr}$. Classification accuracy *ϕ*^*Acc*^ is subsequently calculated based on the consensus of predicted labels *ℓ*(*c*_*i*_) of the Random Forest classifier on the independent testing pool $c_{i}\in E_{ts}$.

### Evaluation of Object Class Separation via Silhouette Index (*ϕ*^*SI*^)

Silhouette Index (SI) offers an independent measure to quantify the separation of multiple classes in the embedding. SI can detect more subtle changes in the embedding with regards to overall class separation compared to classification accuracy. The Silhouette Index (*ϕ*^*SI*^) [58] is a cluster validity measure which jointly takes into account (1) the compactness of samples belonging to the same object class (*ℓ*(*c*_*i*_) = *ℓ*(*c*_*j*_)) and (2) the separation of samples belonging to different object classes (*ℓ*(*c*_*i*_)≠*ℓ*(*c*_*j*_)). The intra-cluster compactness is measured by *A*_*i*_ = ∑_*j*,*ℓ*(*c*_*j*_) = *ℓ*(*c*_*i*_)_‖**y**_*i*_ − **y**_*j*_‖_2_, which represents the average distance of a sample *c*_*i*_ from other samples *c*_*j*_ of the same class in *Y*. Whereas, inter-cluster separation is measured by *B*_*i*_ = ∑_*j*,*ℓ*(*c*_*j*_)≠*ℓ*(*c*_*i*_)_‖**y**_*i*_ − **y**_*j*_‖_2_, the minimum of the average distances of a sample *c*_*i*_ from other samples in different classes. Thus, the formulation for *ϕ*^*SI*^ is as follows,

$$\begin{array}{c} \phi^{SI}=\sum_{i}^{N}\frac{B_{i}-A_{i}}{\max[A_{i},B_{i}]}. \end{array}$$ (10)

*ϕ*^*SI*^ ranges from -1 to 1, where -1 demonstrates the worst, and 1 is the best possible embedding. For each experiment, *ϕ*^*SI*^ is calculated using all labels *ℓ*(*c*_*i*_), $c_{i}\in E_{tr}$ in *Y*.

### Evaluation of Embedding Variance via Classification Accuracy (*ρ*^*Acc*^)

The rate of learning is affected by the initial training examples *S*_*tr*_ provided to the algorithm. It is anticipated that active learning will be able to consistently identify training instances, *S*_*a*_, which will lead to improved classification, whereas random sampling will show more varied improvement due to the variance in the specific training instances chosen. We test the variance in *ϕ*^*Acc*^ of our algorithm (AdDReSS) compared to SSAGE across all runs, each with a unique random initializations *S*_*tr*_. Classification Variance is computed as

$$\begin{array}{c} \rho^{Acc}=\frac{\sum_{i}^{n}{\left(\phi_{i}^{Acc}-{\bar{\phi }}^{Acc}\right)}^{2}}{n-1}, \end{array}$$ (11)

where *n* = 20, representing the number of random initializations, and ${\bar{\phi }}^{Acc}$ refers to the mean across *n* values of $\phi_{i}^{Acc}$, ${\bar{\phi }}^{Acc}=\frac{1}{n}\sum_{i}^{n}\phi_{i}^{Acc}$. A lower *ρ*^*Acc*^ suggests greater robustness to initialization via a more consistent *ϕ*^*Acc*^.

### Evaluation of Embedding Variance via Silhouette Index (*ρ*^*SI*^)

Similar to *ρ*^*Acc*^, we also aim to quantify the variance of the embedding with regards to the Silhouette Index, which reflects the separability of the two object classes in terms of the Euclidean distance between data points in the embedding *Y*. *ρ*^*SI*^ captures the variance in the embedding *Y* across all runs, each with unique, random initializations, such that Silhouette Variance is computed as

$$\begin{array}{c} \rho^{SI}=\frac{\sum_{i}^{n}{\left(\phi_{i}^{SI}-{\bar{\phi }}^{SI}\right)}^{2}}{n-1}, \end{array}$$ (12)

where *N* = 20, the number of random initializations, and ${\bar{\phi }}^{SI}$ refers to the mean across *n* values of $\phi_{i}^{SI}$, ${\bar{\phi }}^{SI}=\frac{1}{n}\sum_{i}^{n}\phi_{i}^{SI}$. A lower *ρ*^*SI*^ suggests greater robustness to initialization in terms of a more consistent *ϕ*^*SI*^.

### Evaluation of Overall Embedding Learning Rate via Raghavan Efficiency (*ϕ*^*Eff*^)

**Fig 11:**
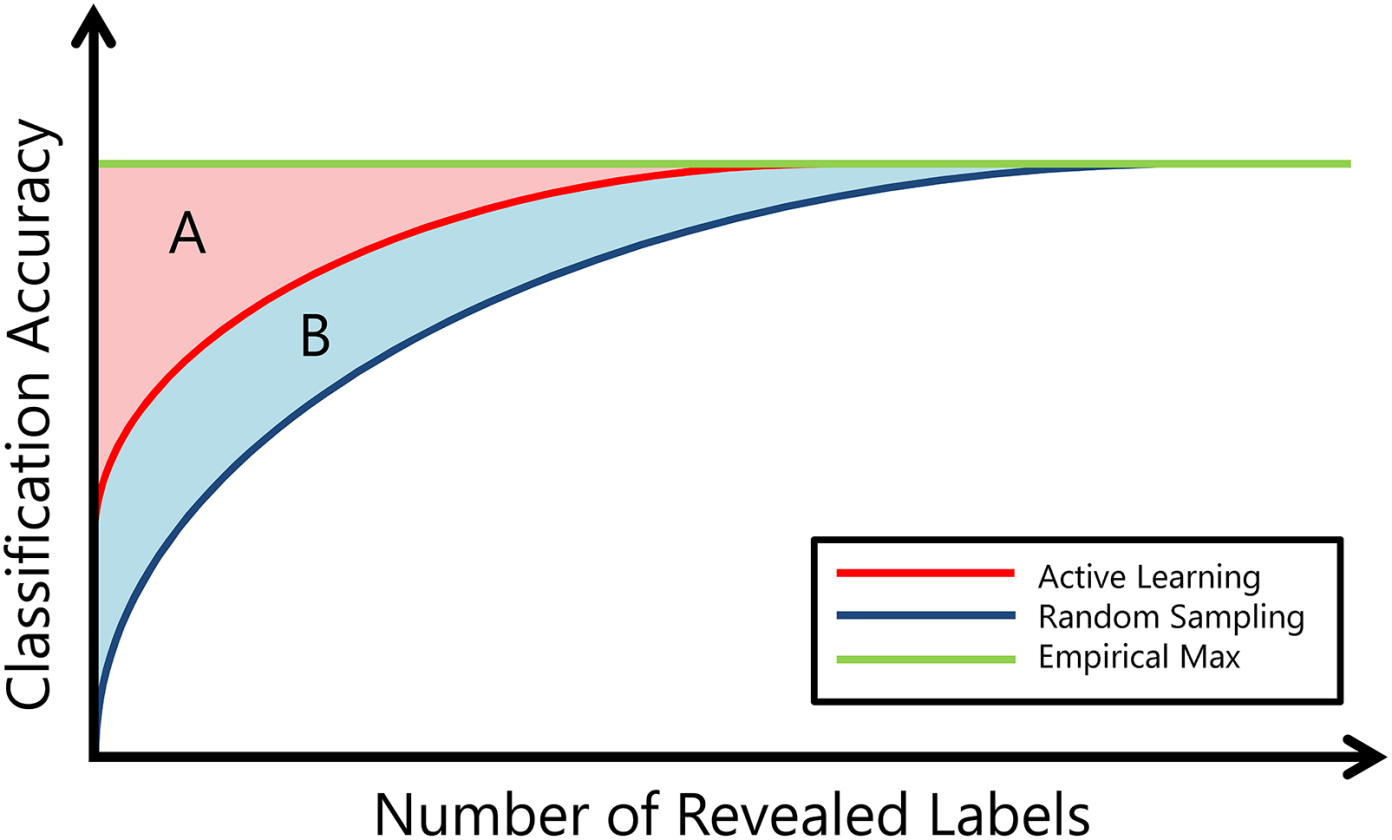
Illustration describing Raghavan efficiency. *A* refers to the area between the Active Learning curve and the empirically-derived maximum accuracy, and *B* refers to the area between the Random Sampling curve and the Active Learning curve.

Raghavan Efficiency [59] describes the rate of learning among active learning algorithms. Fig 11 [46] provides a visual interpretation of Raghavan Efficiency, where the region identified by *A* represents the area between the the Active Learning curve and the maximum achievable performance, and the region defined by *B* represents the area between the the Active Learning curve and the Random Sampling curve. Raghavan Efficiency is defined by a subtraction of the ratio *A*/*B* such that *ϕ*^*Eff*^ ranges between 0 and 1 and larger values of *ϕ*^*Eff*^ are indicative of a faster learning rate. We use *ϕ*^*Eff*^ to compare the overall learning rate between 1) AdDReSS vs GE, 2) SSAGE vs GE and 3) AdDReSS vs SSAGE.

To compare the efficiency of an active learner *Y*^*Ac*^ against random sampling *Y*^*Rd*^, *ϕ*^*Eff*^ may be expressed as

$$\begin{array}{ccc} \phi^{Eff}\left(Y^{Ac}|Y^{Rd}\right) & & =1-\frac{A}{A+B}\\ & & =1-\frac{\sum_{t=t_{0}}^{t_{f}}\phi^{Acc}\left(Y_{l=t_{f}}^{Rd}\right)-\phi^{Acc}\left(Y_{l=t}^{Ac}\right)}{\sum_{t=t_{0}}^{t_{f}}\phi^{Acc}\left(Y_{l=t_{f}}^{Rd}\right)-\phi^{Acc}(Y_{l=t}^{Rd})}, \end{array}$$ (13)

where *t*_0_ and *t*_*f*_ represent the number of initial training samples used to learn *Y*, and the final number of training samples used to learn *Y*, respectively. The empirical maximum accuracy refers to the highest *ϕ*^*Acc*^ obtained for any single iteration of *Y* such that $\phi^{EM}=\max_{i,l}[\phi_{i}^{Acc}\left(Y_{l}^{Ac}\right)]$, where *i* ∈ {1, 2, …, *n*} denotes specific run of *Y*^*Ac*^ with a unique initial training set *S*_*ts*_.

Additionally, to compare AdDReSS and SSAGE against the same baseline comparison, GE, we summarized these results using percentage comparison between for 1) *ϕ*^*Eff*^ (*Y*^*Ad*^|*Y*^*GE*^) and 2) *ϕ*^*Eff*^(*Y*^*SS*^|*Y*^*GE*^). The percentage change in *ϕ*^*Eff*^ for AdDReSS from SSAGE can be expressed as

$$\begin{array}{c} \Delta \phi^{Eff}=(1-\frac{\phi^{Eff}\left(Y^{Ad}|Y^{GE}\right)}{\phi^{Eff}\left(Y^{SS}|Y^{GE}\right)})\times 100\%. \end{array}$$ (14)

## References

[1] Madabhushi A, Agner S, Basavanhally A, Doyle S, Lee G. Computer-aided prognosis: Predicting patient and disease outcome via quantitative fusion of multi-scale, multi-modal data. Computerized medical imaging and graphics. 2011;35(7):506–514. 10.1016/j.compmedimag.2011.01.008 21333490

[2] Lao Z, Shen D, Xue Z, Karacali B, Resnick SM, Davatzikos C. Morphological classification of brains via high-dimensional shape transformations and machine learning methods. Neuroimage. 2004;21(1):46–57. 10.1016/j.neuroimage.2003.09.027 14741641

[3] Yeoh EJ, Ross ME, Shurtleff SA, Williams WK, Patel D, Mahfouz R, et al Classification, Subtype Discovery, and Prediction of Outcome in Pediatric Acute Lymphoblastic Leukemia by Gene Expression Profiling. Cancer Cell. 2002;1(2):133–143. 10.1016/S1535-6108(02)00032-6 12086872

[4] Petricoin EF, Ardekani AM, Hitt BA, Levine PJ, Fusaro VA, Steinberg SM, et al Use of proteomic patterns in serum to identify ovarian cancer. The Lancet. 2002;359(9306):572–577. 10.1016/S0140-6736(02)07746-2 11867112

[5] Doyle S, Monaco J, Feldman M, Tomaszewski J, Madabhushi A. An active learning based classification strategy for the minority class problem: application to histopathology annotation. BMC Bioinformatics. 2011;12:424 10.1186/1471-2105-12-424 22034914 PMC3284114

[6] Geurts P, Fillet M, De Seny D, Meuwis MA, Malaise M, Merville MP, et al Proteomic mass spectra classification using decision tree based ensemble methods. Bioinformatics. 2005;21(14):3138–3145. 10.1093/bioinformatics/bti494 15890743

[7] Hoyle DC. Automatic PCA dimension selection for high dimensional data and small sample sizes. Journal of Machine Learning Research. 2008;9(12):2733–2759.

[8] Singh D, Febbo PG, Ross K, Jackson DG, Manola J, Ladd C, et al Gene expression correlates of clinical prostate cancer behavior. Cancer Cell. 2002;1(2):203–209. 10.1016/S1535-6108(02)00030-2 12086878

[9] Bellman RE. Adaptive Control Processes. Princeton University Press; 1961.

[10] Hughes G. On the mean accuracy of statistical pattern recognizers. Information Theory, IEEE Transactions on. 1968;14(1):55–63. 10.1109/TIT.1968.1054102

[11] Duda RO, Hart PE, Stork DG. Pattern Classification (2nd ed Wiley; 2000.

[12] Dawson K, Rodriguez RL, Malyj W. Sample phenotype clusters in high-density oligonucleotide microarray data sets are revealed using Isomap, a nonlinear algorithm. BMC Bioinformatics. 2005;6:195 10.1186/1471-2105-6-195 16076401 PMC1189082

[13] Lee G, Rodriguez C, Madabhushi A. Investigating the Efficacy of Nonlinear Dimensionality Reduction Schemes in Classifying Gene and Protein Expression Studies. IEEE Trans on Computational Biology and Bioinformatics. 2008;5(3):368–384. 10.1109/TCBB.2008.36 18670041 PMC2562675

[14] Guyon I, Elisseeff A. An introduction to variable and feature selection. The Journal of Machine Learning Research. 2003;3:1157–1182.

[15] Tibshirani R. Regression shrinkage and selection via the lasso. Journal of the Royal Statistical Society Series B (Methodological). 1996; p. 267–288.

[16] Chandrashekar G, Sahin F. A survey on feature selection methods. Computers & Electrical Engineering. 2014;40(1):16–28. 10.1016/j.compeleceng.2013.11.024

[17] Liu M, Zhang D. Pairwise Constraint-Guided Sparse Learning for Feature Selection. Cybernetics, IEEE Transactions on. 2016;46(1):298–310. 10.1109/TCYB.2015.2401733 26151948

[18] Han Y, Yang Y, Yan Y, Ma Z, Sebe N, Zhou X. Semisupervised feature selection via spline regression for video semantic recognition. Neural Networks and Learning Systems, IEEE Transactions on. 2015;26(2):252–264. 10.1109/TNNLS.2014.2314123 25608288

[19] Hotelling H. Analysis of a complex of statistical variables into principal components. Journal of educational psychology. 1933;24(6):417 10.1037/h0071325

[20] Venna J, Kaski S. Local multidimensional scaling. Neural Networks. 2006;19:889–899. 10.1016/j.neunet.2006.05.014 16787737

[21] Cox TFCMAA. Multidimensional Scaling. Chapman and Hall.; 2001.

[22] Scholkopf B, Mika S, Smola A, Ratsch G, Muller KR. Kernel PCA Pattern Reconstruction via Approximate Pre-Images. 1998;.

[23] Shi J, Malik J. Normalized Cuts and Image Segmentation. IEEE Trans Pattern Analysis and Machine Intelligence. 2000;22(8):888–905. 10.1109/34.868688

[24] Tenenbaum J, de Silva V, et al A Global Geometric Framework for Nonlinear Dimensionality Reduction. Science. 2000;290(5500):2319–2322. 10.1126/science.290.5500.2319 11125149

[25] Roweis S, Saul L. Nonlinear dimensionality reduction by locally linear embedding. Science. 2000;290(5500):2323–2326. 10.1126/science.290.5500.2323 11125150

[26] Nilsson J, Fioretos T, Hoglund M, Fontes M. Approximate geodesic distances reveal biologically relevant structures in microarray data. Bioinformatics. 2004;20(6):874–880. 10.1093/bioinformatics/btg496 14752004

[27] Hou C, Nie F, Wu Y. Semi-supervised dimensionality reduction via harmonic functions In: Modeling Decision for Artificial Intelligence. Springer; 2011 p. 91–102.

[28] Golugula A, Lee G, Master SR, Feldman MD, Tomaszewski JE, Speicher DW, et al Supervised regularized canonical correlation analysis: integrating histologic and proteomic measurements for predicting biochemical recurrence following prostate surgery. BMC Bioinformatics. 2011;12(1):483 10.1186/1471-2105-12-483 22182303 PMC3267835

[29] Qian B, Davidson I. Semi-Supervised Dimension Reduction for Multi-Label Classification In: AAAI; 2010.

[30] Shi X, Guo Z, Lai Z, Yang Y, Bao Z, Zhang D. A framework of joint graph embedding and sparse regression for dimensionality reduction. Image Processing, IEEE Transactions on. 2015;24(4):1341–1355. 10.1109/TIP.2015.2405474 25706635

[31] Zhao M, Zhang Z, Chow TW. Trace ratio criterion based generalized discriminative learning for semi-supervised dimensionality reduction. Pattern Recognition. 2012;45(4):1482–1499. 10.1016/j.patcog.2011.10.008

[32] Huang Y, Xu D, Nie F. Semi-supervised dimension reduction using trace ratio criterion. Neural Networks and Learning Systems, IEEE Transactions on. 2012;23(3):519–526. 10.1109/TNNLS.2011.2178037 24808556

[33] Sugiyama M, Idé T, Nakajima S, Sese J. Semi-supervised local Fisher discriminant analysis for dimensionality reduction. Machine learning. 2010;78(1-2):35–61. 10.1007/s10994-009-5125-7

[34] Yang X, Fu H, Zha H, Barlow J. Semi-supervised nonlinear dimensionality reduction. International Conference on Machine Learning. 2006; p. 1065–1072.

[35] Zhao H. Combining labeled and unlabeled data with graph embedding. Neurocomputing. 2006;69(16-18):2385–2389. 10.1016/j.neucom.2006.02.010

[36] Zhang D, et al. Semi-Supervised Dimensionality Reduction. In: SIAM International Conference on Data Mining; 2007.

[37] Verbeek JJ, Vlassis N. Gaussian fields for semi-supervised regression and correspondence learning. Pattern Recognition. 2006;39(10):1864–1875. 10.1016/j.patcog.2006.04.011

[38] Chen Y, Mani S, Xu H. Applying active learning to assertion classification of concepts in clinical text. J Biomed Inform. 2012;45(2):265–272. 10.1016/j.jbi.2011.11.003 22127105 PMC3306548

[39] Freund Y, Seung HS, Shamir E, Tishby N. Selective sampling using the query by committee algorithm. Machine learning. 1997;28(2-3):133–168. 10.1023/A:1007330508534

[40] Liu Y. Active Learning with Support Vector Machine Applied to Gene Expression Data for Cancer Classification. J Chem Inf Comput Sci. 2004;44 (6):1936–1941. 10.1021/ci049810a 15554662

[41] Lee G, Madabhushi A. Semi-supervised graph embedding scheme with active learning (SSGEAL): classifying high dimensional biomedical data In: Pattern Recognition in Bioinformatics. Springer; 2010 p. 207–218.

[42] Zhang L, Chen C, Bu J, Cai D, He X, Huang TS. Active Learning Based on Locally Linear Reconstruction. IEEE Trans Pattern Analysis and Machine Intelligence. 2011; 10.1109/TPAMI.2011.20 21282854

[43] Roux L, Racoceanu D, Loménie N, Kulikova M, Irshad H, Klossa J, et al Mitosis detection in breast cancer histological images An ICPR 2012 contest. Journal of pathology informatics. 2013;4 10.4103/2153-3539.112693 PMC3709417 23858383

[44] Kwan RK, Evans AC, Pike GB. MRI simulation-based evaluation of image-processing and classification methods. IEEE Trans Med Imaging. 1999;18(11):1085–1097. 10.1109/42.816072 10661326

[45] Cortes C, Vapnik V. Support-vector networks. Machine learning. 1995;20(3):273–297. 10.1023/A:1022627411411

[46] Baram Y, El-Yaniv R, Luz K. Online choice of active learning algorithms. The Journal of Machine Learning Research. 2004;5:255–291.

[47] Seung HS, Opper M, Sompolinsky H. Query by committee In: Proceedings of the fifth annual workshop on Computational learning theory. ACM; 1992 p. 287–294.

[48] Hsu CW, Chang CC, Lin CJ, et al. A practical guide to support vector classification. 2003;.

[49] Schohn G, Cohn D. Less is more: Active learning with support vector machines In: ICML; 2000 p. 839–846.

[50] Tong S, Koller D. Support vector machine active learning with applications to text classification. The Journal of Machine Learning Research. 2002;2:45–66.

[51] Chang H, Loss LA, Parvin B. Nuclear segmentation in H and E sections via multi-reference graph-cut (MRGC). In: International Symposium Biomedical Imaging; 2012.

[52] Otsu N. A threshold selection method from gray-level histograms. Automatica. 1975;11(285-296):23–27.

[53] Romo D, García-Arteaga JD, Arbeláez P, Romero E. A discriminant multi-scale histopathology descriptor using dictionary learning In: SPIE Medical Imaging. International Society for Optics and Photonics; 2014 p. 90410Q–90410Q.

[54] Haralick R, Shanmugam K, Dinstein I. Textural Features for Image Classification. IEE Transactions on Systems, Man and Cybernetics. 1973;3(6):610–621. 10.1109/TSMC.1973.4309314

[55] Madabhushi A, Shi J, Rosen M, Tomaszeweski JE, Feldman MD. Graph Embedding to Improve Supervised Classification and Novel Class Detection: Application to Prostate Cancer In: MICCAI; 2005 p. 729–737. 10.1007/11566465_90 16685911

[56] Herlidou-Meme S, Constans JM, Carsin B, Olivie D, Eliat PA, Nadal-Desbarats L, et al MRI texture analysis on texture test objects, normal brain and intracranial tumors. Magnetic Resonance Imaging. 2003;21(9):989–993. 10.1016/S0730-725X(03)00212-1 14684201

[57] Ho TK. The Random Subspace Method for Constructing Decision Forests. IEEE Trans on Pattern Analysis and Machine Intelligence. 1998;20(8):832–844. 10.1109/34.709601

[58] Rousseeuw P. Silhouettes: a graphical aid to the interpretation and validation of cluster analysis. Journal of Computational and Applied Mathematics. 1987;20(1):53–65. 10.1016/0377-0427(87)90125-7

[59] Raghavan H, Madani O, Jones R. Active learning with feedback on features and instances. The Journal of Machine Learning Research. 2006;7:1655–1686.

